# Intracerebroventricular infusion of exercise donor plasma exosomes induces molecular changes indicating exercise-like adaptations in basal ganglia cells of older male rats

**DOI:** 10.3389/fnagi.2026.1766698

**Published:** 2026-05-28

**Authors:** Bruce A. Citron, Kathleen E. Murray, Alexander Lemenze, Anya E. Mausoof, Kevin D. Beck, Fernando J. Velloso, Steven W. Levison, Vedad Delic

**Affiliations:** 1 Laboratory of Molecular Biology, VA New Jersey Health Care System, East Orange, NJ, United States; 2 Department of Pharmacology, Physiology, & Neuroscience, Rutgers New Jersey Medical School, Newark, NJ, United States; 3 Department of Pathology, Immunology, and Laboratory Medicine, Rutgers New Jersey Medical School, Newark, NJ, United States; 4 Center for Immunity and Inflammation, Rutgers New Jersey Medical School, Newark, NJ, United States; 5 NeuroBehavioral Research Laboratory, VA New Jersey Health Care System, East Orange, NJ, United States

**Keywords:** aging, basal ganglia, blood plasma exosomes, exercise, Parkinson’s disease, rat - brain, single nuclei RNA sequencing

## Abstract

Chronic moderate aerobic exercise promotes health in older adults and provides neuroprotection to patients with neurodegenerative diseases, most notably Parkinson’s disease (PD). Exosomes are small extracellular vesicles that facilitate interorgan communication. During exercise, they are selectively packaged with bioactive molecules termed “exerkines” and released into the blood. Exosomes protect their exerkine cargo, including DNA, RNA, and proteins, and facilitate their interorgan transit. Since aging is the primary risk factor for common neurodegenerative disorders, including PD, in this study, we explored the potential to transfer exercise-induced adaptive changes from active donors to sedentary recipients via exosomal factors in aged rats. We hypothesized that infusing exosomes from exercised donors could induce exercise-like changes in the brains of sedentary recipients. Aged sedentary rats fitted with an intracerebroventricular cannula were infused with plasma exosomes from exercised or sedentary donors. The basal ganglia were removed for single-nuclei RNA sequencing and immunoblotting. When compared to controls, rats that received exosomes from exercised donors had lower gene expression associated with activated microglia, as evidenced by decreased mTOR signaling and oxidative phosphorylation. Parkinson’s disease signaling pathways were most significantly downregulated in astrocytes, while calcium, cyclic adenosine monophosphate (cAMP), and mitogen-activated protein kinase (MAPK) signaling pathways were downregulated in microglia. Neuronal activity was mixed; for example, in dopaminergic neurons of the substantia nigra, neuroactive ligand–receptor interaction and calcium signaling pathways were upregulated, while oxidative phosphorylation was downregulated. The levels of exercise-associated peptides brain-derived neurotrophic factor (BDNF) and irisin were also elevated. This proof of concept study demonstrates that the infusion of exosomes from the plasma of exercised donors elicits cell-specific molecular changes consistent with exercise-induced adaptations in the brains of sedentary recipients.

## Introduction

Brain changes associated with aging are both morphological and molecular, resulting in reduced total brain volume and vascular remodeling that together predispose the brain to stroke and neurodegenerative diseases (Peters, 2006). Advancing age is the primary risk factor for most common neurodegenerative disorders, including Alzheimer’s disease (AD) and PD (Hou et al., 2019). Brains of asymptomatic elderly individuals often have higher levels of protein deposits, such as hyperphosphorylated tau (p-tau) and amyloid-β (Aβ) found in AD, and aggregated alpha-synuclein protein, a hallmark of PD (Elobeid et al., 2016). Accumulation of these pathologies can counter the robust molecular homeostasis necessary to maintain normal brain function.

The onset of neurodegenerative changes involves several processes affecting both glia and neuronal cells. Neurons are the most energy-demanding cell type in the body and rely on large numbers of mitochondria for their metabolic needs (Misgeld and Schwarz, 2017). However, mitochondria are also the site of most intracellular reactive oxygen species (ROS) production, and ROS damage neuronal membranes, nucleic acids, and intracellular proteins (Palma et al., 2024). To cope with the stressors of long lifespan, high-energy demand, and the resultant damage accumulation, neurons exhibit robust molecular homeostasis to maintain long-lived nucleic acids and proteins (Hetzer and Toda, 2025). Nevertheless, the vigorous neuronal machinery that maintains homeostasis for decades eventually falters and fails with age, resulting in senescence.

Glial cells support neurons by detoxifying excitatory neurotransmitters such as glutamate, maintaining ionic and pH homeostasis, neutralizing ROS, and providing nutrients and neurotrophic factors. With advancing age, glial cells have the propensity to progress toward a more reactive state termed “inflammaging,” where they assume a less trophic and a more reactive pro-inflammatory state, leaving aging neurons in a precarious state (Franceschi et al., 2000). This pro-inflammatory state can be further exacerbated by exposure to toxicants or injury over a lifetime, resulting in accelerated neuronal death and the onset of symptoms associated with neurodegenerative diseases, particularly PD (Delic et al., 2020). Indeed, there exists a spectrum of pathology burden in aged brains from asymptomatic to symptomatic PD (Calabrese et al., 2018).

While aging is considered a largely inevitable and irreversible process, beneficial lifestyle changes such as diet and exercise can slow the biological aging process, reduce the risk of neurodegenerative diseases, and even inhibit disease progression. Indeed, cardiorespiratory fitness that can be improved by exercise is the strongest predictor of a healthy brain later in life (Wittfeld et al., 2020; Oberlin et al., 2025). Exercise studies demonstrating neuroprotection have shown that structured, repetitive, challenging, and chronic exercise—performed regularly for months and years—improves outcomes in PD patients (Tsukita et al., 2022). In animal models of PD, exercise has been shown to slow neurodegeneration (Chen et al., 2018; Citron et al., 2025). At levels of exercise that provide neuroprotection in a preclinical PD model, we recently identified multiple mRNAs with neuroprotective potential that were significantly upregulated in plasma exosomes of chronic exercisers (heretofore referred to as eExosomes) (Citron et al., 2025). Exosomes are small extracellular vesicles (EVs), smaller than 200 nm, and have indicators of subcellular origin (Welsh et al., 2024). Our isolated EVs met these exosome criteria, ranging in size from 30 nm to less than 150 nm, and were CD63 positive, a marker of late endosomal multivesicular body origin (Sung et al., 2020). Isolated exosomes were also positive for microRNAs typically present in skeletal muscle, suggesting enrichment for skeletal muscle as the tissue of origin. We wanted to determine if the basal ganglia, a subcortical brain region containing multiple nuclei affected primarily in PD, undergoes exercise-adaptive changes in response to exercise exosomes. These results could help explain the neuroprotective benefits of direct exercise in a PD model we previously reported (Citron et al., 2025). Exosomes have been shown to cross the blood–brain barrier (Banks et al., 2020) and likely do so during exercise; however, the rate at which they can cross the BBB and the length of time required to induce exercise-like changes are unknown. To overcome these technical challenges, as a proof of concept, delivery of exosomes was planned through intracerebroventricular (ICV) infusion for maximum effect.

To test our hypothesis that eExosomes, abundant in the plasma of chronic exerciser rats, can communicate neuroprotective adaptive changes to the brain, eExosomes isolated from exercised donor plasma were infused by ICV once daily for 7 days into sedentary aged rats. Single-nuclei RNAseq was performed, followed by bioinformatic analyses to determine the degree of gene expression changes induced by exosomes. Peptide levels of irisin and BDNF—both known to be upregulated in the brain with exercise—were also measured and reported herein (Villamil-Parra and Moscoso-Loaiza, 2024).

## Methods

### Animal usage

**Figure 1:**
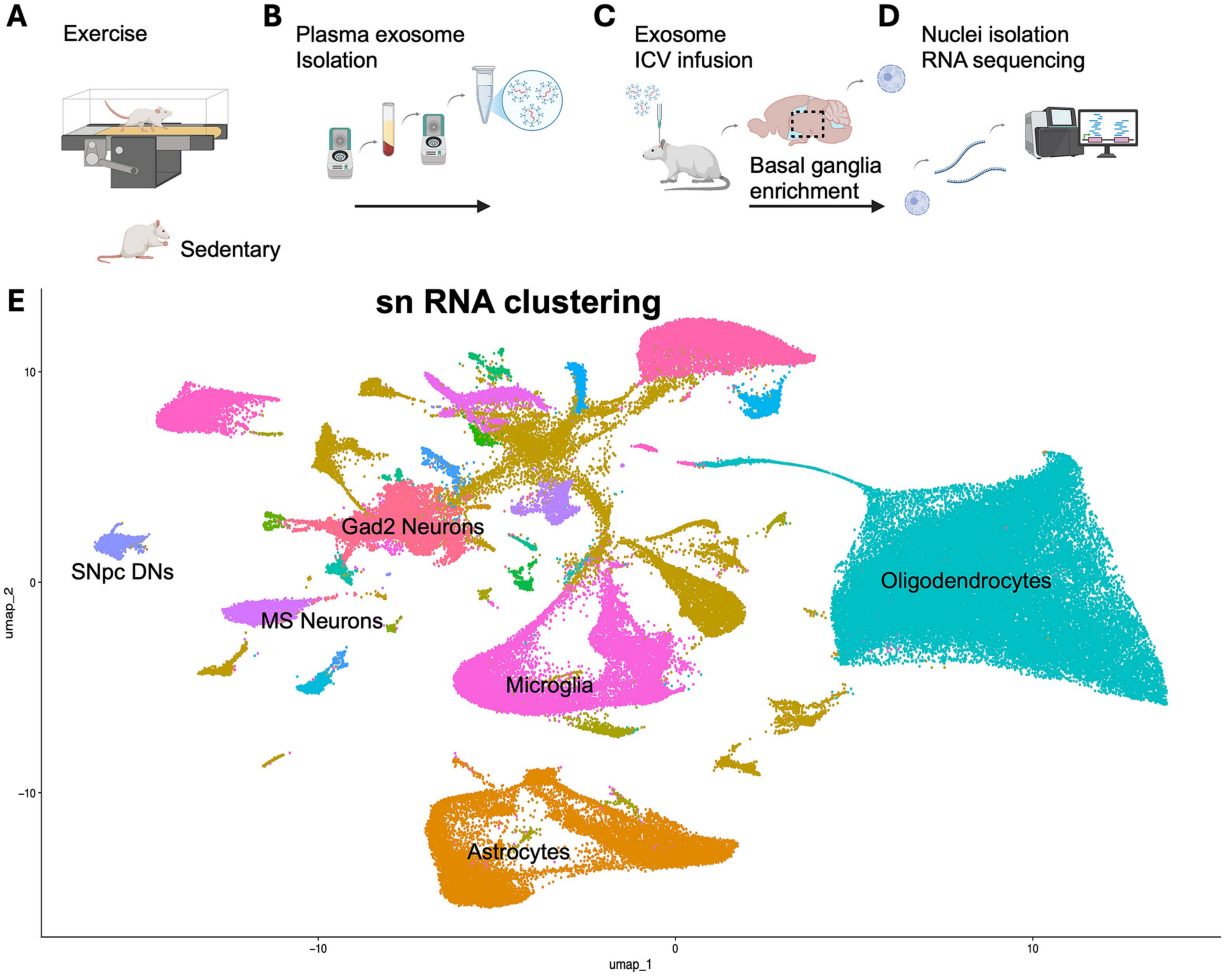
Workflow and cluster identification. Ten-week-old male Sprague Dawley (SD) rats were obtained from Charles River, of which 5 were randomly assigned to the sedentary group and 5 were assigned to the exercise donor groups. Exercise donor rats underwent an exercise regimen 5 days a week for 1 month **(A)**. After the last bout of exercise, rats were exsanguinated and plasma exosomes isolated **(B)**. 11-month-old male rats (retired breeders) were fitted unilaterally on the left anatomical side of the calvarium with an intracerebroventricular (ICV) cannula by Charles River and infused daily for 7 days with 48 μg of exercise or sedentary donor plasma exosomes in 10 μL of PBS under light anesthesia. After the last infusion, the rats were euthanized and perfused with ice-cold saline. Brains were extracted, and a region enriched for basal ganglia was dissected **(C)**. Tissue was rapidly frozen on dry ice. For single-nucleus RNA-seq, the tissue was pulverized under liquid nitrogen using a mortar and pestle kept on dry ice. The samples were then processed to isolate nuclei. Isolated nuclei were validated with flow cytometry and sequenced **(D).** A total of 26 distinct cell clusters were identified, including cells of primary interest with their cell counts per cluster: Astrocytes 16,079, dopaminergic neurons from substantia nigra pars compacta 1,591, Gad2 neurons 8,284, medium spiny neurons 2,343, microglia 12,529, and oligodendrocytes 45,600 **(E)**. Workflow diagram was created with BioRender.

Exosomes were isolated from exercise or sedentary donors and infused into old sedentary recipients, followed by extraction of basal ganglia, nuclei isolation, and RNA sequencing (Figures 1A–D). A total of 10 retired male Sprague Dawley (SD) breeders (11 months of age) were obtained with pre-implanted intracerebroventricular (ICV) cannulas from Charles River Laboratories (Wilmington, MA) and handled in accordance with the VA New Jersey Health Care System Institutional Animal Care and Use Committee (IACUC) guidelines. Cannulated rats were randomly assigned to exercised or sedentary exosome recipient groups. Additional male SD rats aged 9–10 weeks (Charles River) were obtained for exosome harvest and were randomly assigned to sedentary or exercise donor groups. The rats were housed individually in standard polycarbonate 18-quart tubs with Bed-o’-Cob bedding that was changed once a week. Rooms were kept at 22 ± 4 °C, and the rats were housed on a 12-h-on and 12-h-off reverse light cycle to allow for experiments during rat subject active phases. One rat assigned to the exercised exosome recipient group perished before the first treatment. To minimize stress during handling and experiments, rats were acclimated to the experimenter by handling for a week before the experiments were performed. For euthanasia, rats were deeply anesthetized with 5% v/v of isoflurane delivered at a rate of 1.5 L/min of oxygen for 5 min. Full anesthesia was confirmed through the absence of hindlimb reflex. Exosome infused rats underwent a bilateral thoracotomy, followed by perfusion with ice–cold saline by severing the right atrium and infusing through the left ventricle. The brain was collected and dissected on ice to examine the basal ganglia. Similarly, exosome donor rats were anesthetized and exsanguinated by cardiac puncture in the left ventricle after a bilateral thoracotomy.

### Exercise

eExosome male donor rats aged 9–10 weeks were continuously monitored while running on a 5-lane treadmill that was equipped with both a light signal and a tail shock (MazeEngineers, Skokie, IL). Up to 50% attrition was observed at speeds greater than 20 m/min for 30 min. Therefore, exercised rats underwent a warm-up for 5 min at 10 m/min, followed by 30 min at 20 m/min 4–5 days per week for 1 month. Sedentary controls were also 9–10-week-old male rats obtained from the same vendor and were randomly assigned to sedentary groups. Exosomes were obtained from a previous study that characterized their content (Citron et al., 2025).

### Exosomal isolation

Exercise and sedentary exosomes were isolated, and their content was characterized in a parallel study (Citron et al., 2025). Four exercised and five sedentary male donor rats were exsanguinated, and the plasma was collected by cardiac puncture into the left ventricle. The plasma samples from the rats in each group (250 μL from each rat) were pooled for two treatments. Exosomes were isolated from fresh rat plasma with the Total Exosome Isolation kit Invitrogen from plasma (44–844-50), according to the manufacturer’s instructions. Briefly, the pooled plasma samples were centrifuged at 2,000 x g for 20 min at room temperature to eliminate cells and debris. The supernatant was then transferred to a new sterile tube and centrifuged at 10,000 x g for 20 min. Approximately 700 μL of plasma per tube was then transferred to a new tube and placed on ice. To precipitate exosomes, 350 μL of 1x phosphate-buffered saline (PBS) was added and vortexed to mix thoroughly, followed by 210 μL of exosome precipitation reagent. The solution was mixed and then incubated for 10 min at room temperature. The sample was then centrifuged at 10,000 x g for 10 min at room temperature. The supernatant was discarded, and the precipitated exosomes were centrifuged again at 10,000 x g for 1 min. The supernatant was carefully discarded, and each exosome pellet was resuspended in 500 μL of PBS. Isolated plasma exosomes were then assessed for the total number of particles and size using NanoSight (Malvern Panalytical). Particles smaller than 20 nm and greater than 150 nm in diameter were marked red, indicating that they made up a small subset of the total exosome isolate (Citron et al., 2025). For further validation, the samples were subjected to immunoblotting for the exosomal markers CD63. Validation of exosomes was performed along with a concurrent exosome sequencing study (Citron et al., 2025). The exosome suspension was stored at 4 °C and used for 1 week of infusions. Exosome prep was negative for peptides BDNF, irisin, and neuropeptide Y (NPY) present in the blood, as indicated in supplemental immunoblot figures in our companion study (Citron et al., 2025).

### Exosome infusion

Cannulas were placed into the lateral ventricles of 10 recipient, 11-month-old rats for this proof-of-concept study, with the explicit goal of bypassing the blood–brain barrier for maximum dosing effect. Cannulated rats were randomly assigned to a sedentary or exercise exosome recipient group (*n* = 5). Power analysis based on previous RNA sequencing indicated that group sizes of 3, power of 0.90, and alpha of 0.05 were sufficient to detect significant differences with an effect size of 3.59 (Murray et al., 2025). The cannula injection system consisted of a guide cannula, a dummy cannula, and an internal cannula (injector). The 22-gauge guide cannula was implanted using stereotaxic coordinates into the left lateral ventricle. A dummy cannula was inserted into the guide cannula and screwed on top to prevent contamination of the guide cannula. The internal cannula (the injector) was 28 gauge in diameter with an interlocking mechanism with the guide cannula to ensure accurate penetration and depth. Dosing was performed using a Hamilton 700 Series Microliter Syringe (Hamilton Company, Reno, NV, USA) attached to polyethylene (PE) 50 catheter tubing that was securely attached to the injector/internal cannula. Light sedation was induced (typically 5% isoflurane at 2 L/min oxygen for 4 min) and maintained with 4% isoflurane at 1.0 L/min for 4 min. A 10-μl bolus suspension of the exosomes in 1x PBS was infused for a total of 48 μg of protein per day for 7 days.

### Western blots to detect neuroprotective peptides

Basal ganglia enriched brain tissue from exercised and sedentary exosome infused rats were resuspended in radioimmunoprecipitation assay (RIPA) buffer, homogenized, and centrifuged at 4 °C for 5 min at 14,000 rpm to remove cellular debris. Protein concentration was determined using the Pierce™ BCA Protein Assay Kit (ThermoFisher) and iMark™. Absorbance was evaluated using a microplate reader (Bio-Rad Laboratories, Hercules, CA, USA). For Western blot analysis, up to 30 μg of protein was loaded onto 4–20% Mini-PROTEAN TGX Stain-Free Gels (Bio-Rad). Electrophoresis was conducted at 200 V for 30 min. For total protein normalization, before transfer, the stain-free gels were cross-linked using a Bio-Rad ChemiDoc™ imaging system with a UV transilluminator for 1 min. The protein was transferred to a 0.2-μm PVDF membrane at 2.5 A and 25 V for 7 min using a semi-dry Bio-Rad® Trans-Blot Turbo™, blocked using a blotting-grade blocker (Bio-Rad #1706404), and incubated with antibodies to either irisin (Fisher catalog# NB188451 at 1:1000 dilution) or BDNF (Fisher catalog# NC1089906 at 1:1000 dilution). Secondary horseradish peroxidase (HRP) antibodies (Bio-Rad# 0300-0108P, 5,196–2,504 at 1:10000) were then visualized using Clarity Max™ Western ECL substrate (Bio-Rad) and were scanned on the ChemiDoc. This analysis was performed using Image Lab Version 5.2 (Bio-Rad), with values normalized to the total protein visualized using Stain-Free Protein Gels (Bio-Rad). A total number of sedentary and exercise exosome-infused old rats were 5 and *n* = 4, respectively.

### Nuclei isolation and validation

Brain tissue enriched for the basal ganglia was dissected and rapidly frozen in liquid nitrogen. Subsequently, it was pulverized into fine granules using a mortar and pestle on dry ice. Nuclei were isolated following a modified version of the 10X Genomics demonstrated protocol for Nuclei Isolation from Adult Mouse Brain Tissue for Single-Cell RNA Sequencing (CG000393, Rev. A), with all steps performed on ice. Dulbecco’s modified Eagle’s medium (DMEM) made by GIBCO supplemented with 10% FBS (26,140,079, Gibco, Thermo Fisher Scientific, Waltham, MA) was used to resuspend the pulverized tissue in a 50-ml conical tube. Tissue in media was spun down at 100 rcf at 4 °C for 5 min, and the supernatant was reserved. The tissue was then lysed on ice for 15 min in a lysis buffer containing 10 mM Tris–HCl (pH 7.4, T2194, Sigma-Aldrich, St. Louis, MO), 10 mM NaCl (59222C, Sigma-Aldrich), 3 mM MgCl2 (M1028, Sigma-Aldrich), and 0.1% NP-40 (85,124, Thermo Scientific) in ultrapure water. Reserved media was added back, and tissue was triturated using a fire-polished silanized Pasteur pipette and then strained through a 30-μm MACS SmartStrainer (130–098-458, Miltenyi Biotec, Germany) to remove debris. Nuclei were washed three times using nuclei wash buffer containing 1% BSA (Fisher Scientific catalog# MP219989710) and 0.2 U/μl Protector RNase inhibitor (3,335,402,001, Roche, Basel, Switzerland) in 1X PBS without Ca2 + or Mg2 + (21-040-CV, Corning, Corning, NY, USA). For each wash, resuspended tissue was strained, centrifuged at 500 rcf at 4 °C for 5 min, and resuspended in fresh nuclei resuspension buffer at volumes specified in the 10X Genomics protocol and then at a final volume of 1 mL for FACS.

To remove additional debris, nuclei were stained with 1 mg/mL Hoechst 33342 (H3570, Invitrogen, Thermo Fisher Scientific) and sorted on a BD FACSAria II (BD Biosciences, Franklin Lakes, NJ, USA). The samples were acquired by gating for DAPI to identify single G1/S/G2 populations. Single-nuclei purity, yield, and removal of debris were verified by examining the sorted nuclei with the ORFLO Moxi GO II (ORFLO Technologies, Livermore, CA) with a target concentration of 700–1,200 nuclei/ml to proceed with sequencing.

### RNA sequencing

Gene expression libraries were prepared using the Chromium GEM-X Single-Cell 3’ Reagent Kits v4 (10X Genomics, Pleasanton, CA) according to the manufacturer’s protocol (Protocol # CG000731). Briefly, 29,000 flow-sorted nuclei were loaded onto a Chromium GEM-X Single-Cell 3’ Chip for a targeted recovery of 20,000 nuclei, followed by GEM generation and barcoding on a Chromium iX instrument. After GEM-RT incubation, the samples were cleaned up, and cDNA was amplified. The cDNA was then fragmented using an enzymatic reaction, followed by end-repair and A-tailing of cDNA fragments. The fragments were then size-selected, Illumina-compatible adaptors were ligated to the fragments, and indexed libraries were prepared by the sample index PCR reaction. After library preparation and bead purification, each library was diluted to a concentration of 4 nM. The libraries were then pooled together, diluted to a concentration of 2 nM, and sequenced on the NovaSeq X Plus instrument (Illumina, San Diego, California) using the 100-cycle kit to obtain approximately 1 billion reads per sample (approximately 50,000 reads/cell). The libraries were sequenced at a final loading concentration of 170 pM with 1% PhiX according to the Illumina sequencing guide for the NovaSeq X Plus instrument. The following paired-end sequencing configuration was used: Read1: 28 bp, Read2: 90 bp, i7 index: 10 bp, and i5 index: 10 bp. A total number of sedentary and exercise exosome-infused old rats were 5 and 4, respectively.

### Cluster identification

Neuronal clusters were identified with a combination of canonical markers and cluster-unique gene expression. Overall, one to three validated mRNA markers from the literature were used to identify each cluster. Gene expression in each cluster was sorted by the expression of genes in other clusters in ascending order, and fold change expression in the given cluster was listed in descending order. In clusters without canonical gene markers, the top candidate gene marker would have at least 1 characterized cell-specific role, no presence in the other clusters, and a minimum of a 0.5-fold increase in expression compared to the control. The identified cell clusters are listed below, along with gene markers and corresponding references used for identification. For glia, more established biomarkers were available from a single cited source.

*Neuronal markers included* dopaminergic neurons SNpc [Chgb (Zhang K. et al., 2014; Wen et al., 2021)], medium spiny neurons [Drd2 (Skene et al., 2018), Ppp1r1b (Saunders et al., 2018), Pde1b (Zhai et al., 2024)], pyramidal neurons hippocampus dentate gyrus [BDNF (Hofer et al., 1990), Gadd45b (Ma et al., 2009), Fosl2 (Gao et al., 2017)], excitatory neurons cortex [Smpd3 (Stoffel et al., 2018)], CA2 hippocampal pyramidal neurons [Nr3c2 (Viho et al., 2022), Tenm2 (Liakath-Ali et al., 2024)], pyramidal neurons cortex [ENSRNOG00000003742 (Awad et al., 2024)], TRDN neurons [Trdn (van Velthoven et al., 2025)], magnocellular neurosecretory neurons [Caprin2 (Konopacka et al., 2015)], GABAergic interneurons [Vip (Apicella and Marchionni, 2022), Dix1 (Pla et al., 2018), Npas1 (Troppoli et al., 2024)], cholinergic neurons (Kctd8 (Ren et al., 2022)), Gad2 + inhibitory neurons [Nova1 (Tajima et al., 2023), Scn9a (McDermott et al., 2019)], Purkinje cells [Tspoap1 (Mencacci et al., 2021), Ajap1 (Fruh et al., 2024)], corticothalamic projection neurons [Tle4 (Galazo et al., 2023)], Cajal–Retzius cells [Ndnf (Kuang et al., 2010), Grsf1 (Gao G. et al., 2025)], BNC2 neurons in hypothalamus, arcuate nucleus [Bnc2 (Tan et al., 2024)], paraventricular nucleus of the thalamus neurons [Parm1 (Shima et al., 2023), Trhr (Nillni, 2010)], paraventricular and supraoptic nuclei neurons [Sim1 (Duplan et al., 2009)], parvalbumin-positive neurons [Kcns3 (Georgiev et al., 2012)], hippocampus CA3 pyramidal neurons [Rspo2 (Liu et al., 2024)], and angiotensin II receptor type 1a-positive neurons [slc9a4 (Sakuta et al., 2020)]. Since the isolation of the basal ganglia was an enrichment, the presence of some neuronal nuclei from other brain areas, e.g., cortex, was expected. *Astrocyte markers* included Gfap, Slc1a2, Slc1a3, and Aqp4 (Yu et al., 2024). *Microglial markers* included Tmem119, Aif1(IBA1), P2ry12, and Cx3cr1 (Bennett et al., 2016). *Oligodendrocyte markers* included Mog and Mbp (Chamling et al., 2021).

### Statistical analysis

Significance was indicated by * *p* < 0.05 and ** *p* < 0.01. Open bar graphs indicate means with closed circles that represent individual data points, and error bars represent standard deviation.

Raw reads were barcode deconvoluted and aligned with the reference genome (BN7) via Cell Ranger (v9.0.1). All subsequent processing was performed using the Seurat package (v5.2.1) within R (v4.4.2). Low-quality nuclei (nuclei with a percentage of reads of mitochondrial origin >2%, a percentage of reads of ribosomal origin >2%, <100 gene counts, <700 read counts, or MADs >5) were filtered from the dataset, and read counts were normalized using the SCTransform method.^1^ The samples were integrated and clustered via UMAP according to nearest neighbors and annotated based on population markers. Cluster annotations were applied with a combination of SingleR and canonical marker classification. Pseudobulk differential expression between groups was performed via DESeq2 (v1.46) on a per-cluster basis, with differential expression classified as genes with padj < 0.05. Pathway analysis of genes was executed via gene set enrichment analysis (GSEA) of genes ranked by the Wald statistic (clusterProfiler v4.14.4). Gene set enrichment analysis was carried out using genes ranked by the Wald statistic from the differential expression analysis for exercised versus sedentary per cluster. For each gene set, an enrichment score was calculated, and its significance was assessed by generating a null distribution using permutations of gene labels. The nominal *p*-value represents the proportion of permuted enrichment scores that were as extreme as the observed score. Multiple testing correction was performed using the Benjamini–Hochberg procedure to obtain FDR *p*-values.

## Results

### Twenty-six unique clusters, including dopaminergic neurons, astrocytes, microglia, and oligodendrocytes, displayed significant molecular pathway changes

**Figure 2:**
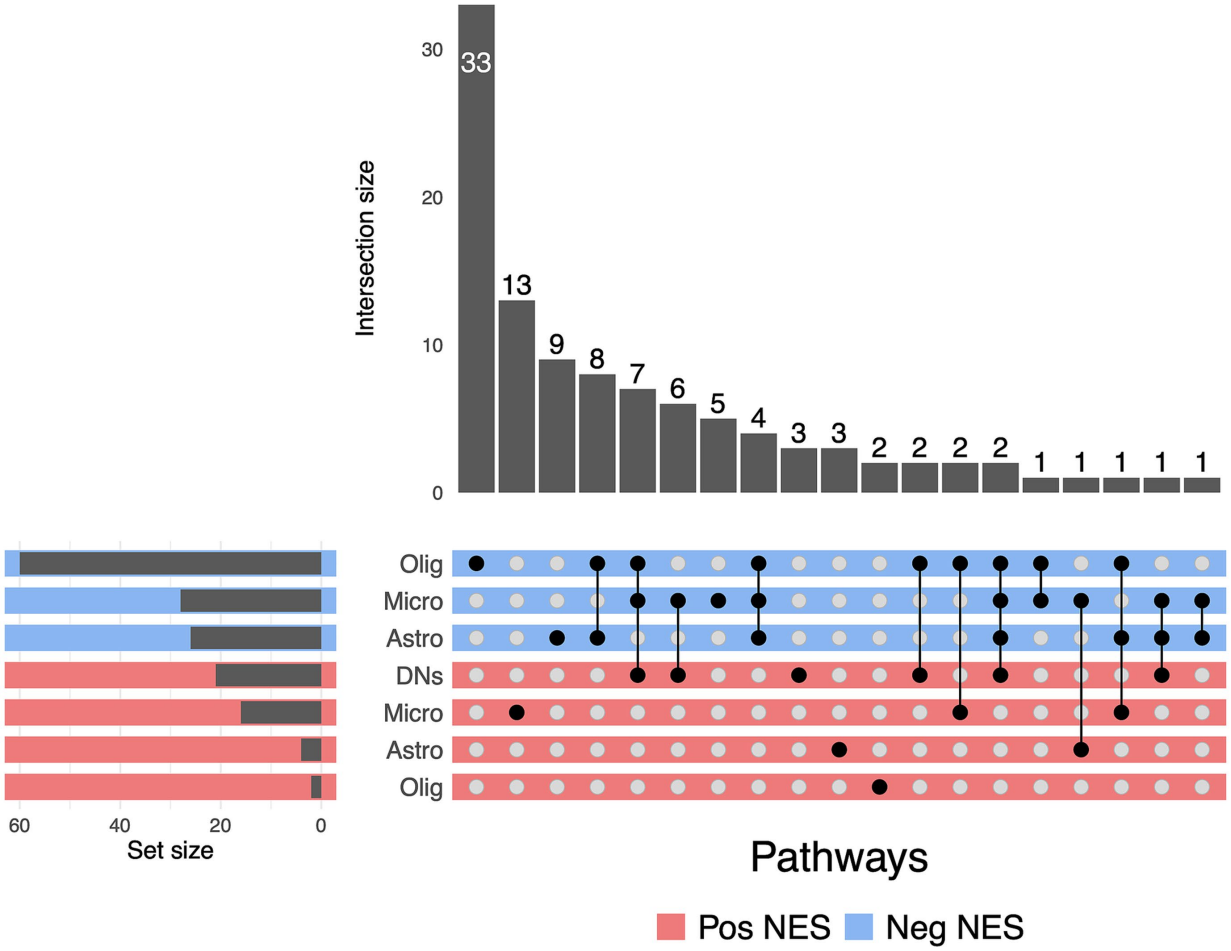
Cell-specific pathway-level expression changes are mostly downregulated in glia and upregulated in dopaminergic neurons. An upset plot displays significant pathway changes in each cell type and whether these pathway changes are shared with other cell types, indicated by the vertical connection lines. The left panel indicates how many pathways are present in that row. Blue indicates downregulated pathways, and red indicates upregulated pathways.

A total of 19 distinct neuronal clusters were identified that included medium spiny neurons of the striatum, GAD2 + interneurons, and dopaminergic neurons of the substantia nigra pars compacta. Glial clusters including astrocytes, microglia, and oligodendrocytes were also identified, along with one unidentified cluster and one multiplet cluster. The entire dataset is uploaded to the National Center for Biotechnology Information (NCBI) Gene Expression Omnibus (GEO) repository. For clarity, the figure is focused on cells of the basal ganglia primarily affected in PD that include astrocytes, dopaminergic neurons of the substantia nigra (SNpc DNs), Gad2 neurons, medium spiny (MS) neurons, oligodendrocytes, and microglia (Figures 1A–E). Infusion of exosomes from exercised rats predominantly caused a downregulation of molecular pathways in glia and an upregulation in neurons (Figure 2). The largest set of identified molecular pathways was in oligodendrocytes, and they were almost entirely downregulated (Figure 2). A considerable overlap of the downregulated pathways was found between astrocytes, microglia, and oligodendrocytes (Figure 2). Some pathways were inversely regulated in glia compared to neurons.

### Exercise-like adaptive gene expression changes in Parkinson’s disease signaling pathways of dopaminergic neurons

**Figure 3:**
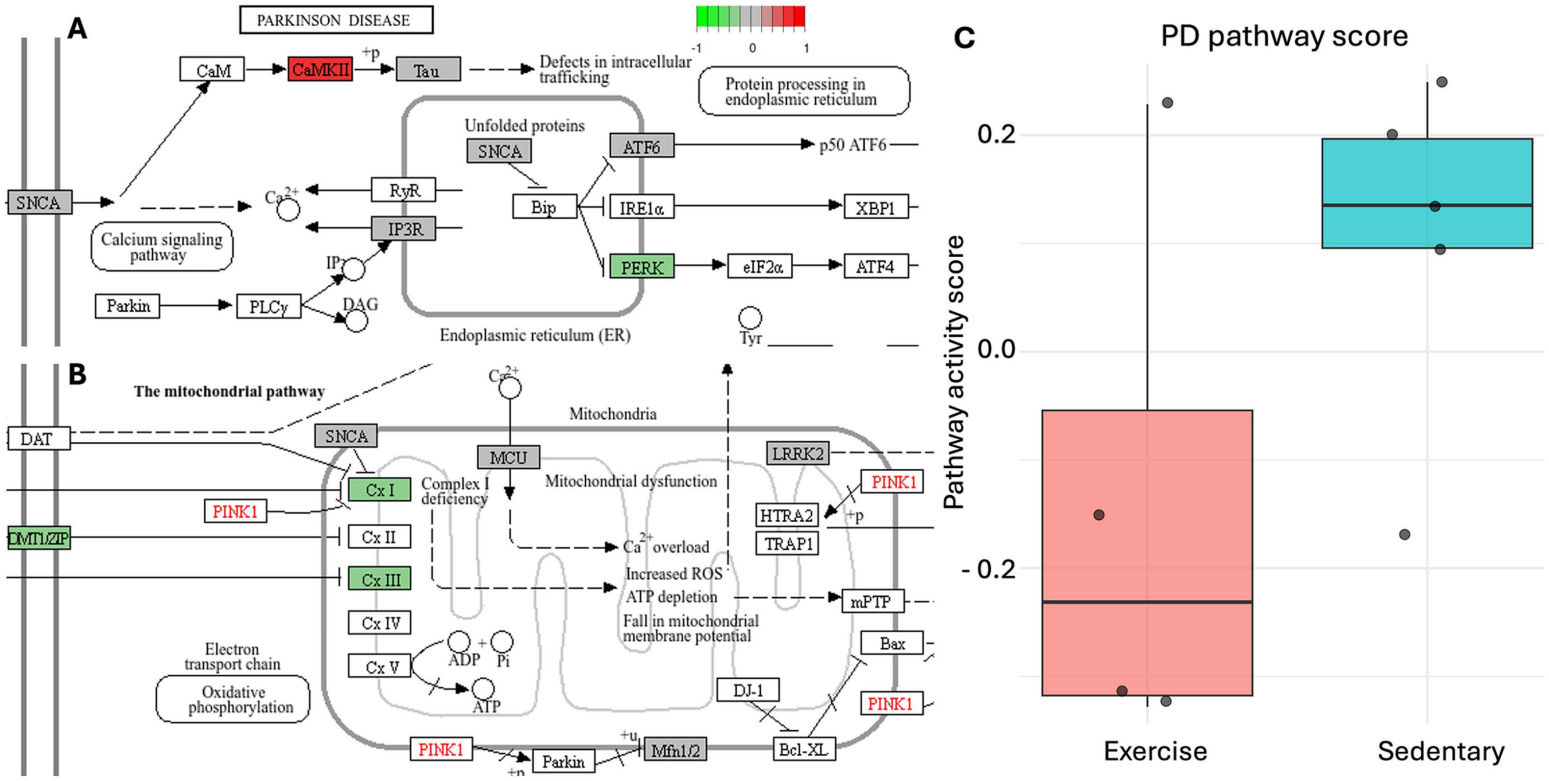
Parkinson’s disease pathway: Increases in calcium signaling and decreases in mitochondrial electron transport chain complexes in dopaminergic neurons. Upregulation of CAMKII signaling and decrease in PERK **(A)**. Decrease in mitochondrial complexes I and III and Divalent Metal Transporter 1 (DMT1) ZRT/IRT-like proteins (ZIP) expression **(B)**. Overall decrease in pathway activity for enzymes affected in Parkinson’s disease **(C)**. Green indicates decrease, and red indicates an increase in PD pathway gene expression score. Gray genes participate in the PD pathway; white box genes do not participate in the pathway. Red text (PINK1) indicates a common mutation in the PD pathway.

**Figure 4:**
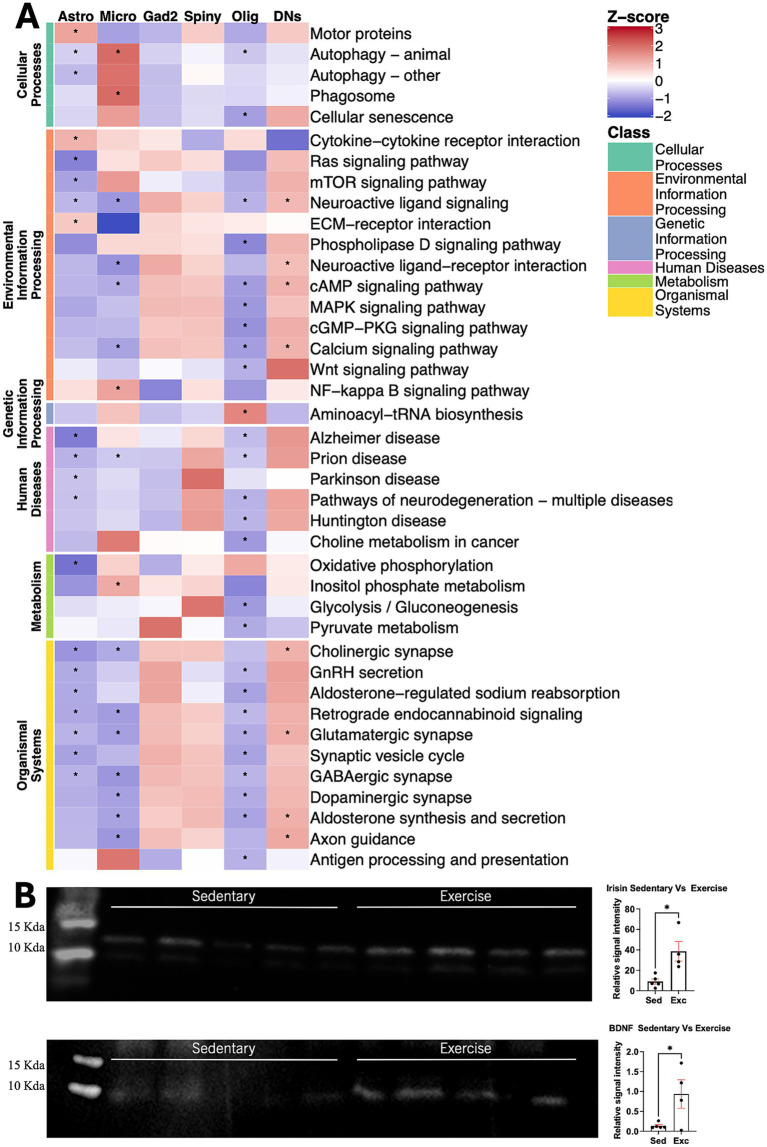
Exercise-adaptive gene and protein-level changes after exosome infusion. Exercise vs. sedentary gene expression changes heat map. Blue indicates downregulation, and red indicates upregulation **(A)**. Western blots for BDNF and irisin in basal ganglia of rats infused with exercise vs. sedentary exosomes despite small sample size **(B)**. Quantification of Western blot data. *n* = 4 (eExosome) and *n* = 5 (sedentary exosome) treated rats. *p < 0.05.

In the brains of eExosome-infused rats, our data revealed decreases in gene expression for complexes I and IV in dopaminergic neurons of the substantia nigra (Figure 3B). Protein kinase R-like endoplasmic reticulum kinase (PERK) is an endoplasmic reticulum protein involved in the unfolded protein response signaling pathway and is activated by ROS (Liu and Zhang, 2020). PERK gene expression was downregulated, which correlated with the downregulation of ROS-producing electron transport complexes I and IV gene expression (Figures 3AB). CaMKII upregulation was expected (Figure 3A), since it was previously reported that exercise elevated activated CaMKII, which can increase BDNF by phosphorylating MeCP2 (Gomez-Pinilla et al., 2011). Indeed, we observed increased levels of BDNF peptide in basal ganglia homogenate (Figure 4B). Divalent Metal Transporter 1 (DMT1) and ZIP are involved in zinc and iron transport regulation and are downregulated with eExosome treatment (Figure 3B). DMT1 is upregulated in the substantia nigra of PD patients, and its inhibition provided neuroprotection (Salazar et al., 2008). Together, these gene expression changes resulted in an overall decrease in PD pathway activity score (Figure 3C). Irisin expression in the brain provides neuroprotection by boosting the expression of BDNF (Lourenco et al., 2019). eExosome treatment caused a significant increase in irisin and BDNF levels (Figure 4B).

### Infusion of exercise donor exosomes resulted in gene and protein level changes associated with adaptation to exercise

A total of 40 pathways were significantly dysregulated across 6 brain cell types, including astrocytes, microglia, oligodendrocytes, dopaminergic neurons in the substantia nigra, Gad2 + GABAergic interneurons, and medium spiny neurons (Figure 4A). Significantly dysregulated molecular pathways included those involved in cellular processes, environmental information processing, genetic information processing, human disease, and metabolism.

Forty-two percent (17) of the pathways in astrocytes were significantly downregulated, while only 7% (3) were upregulated. In microglia, 30% (12) of the pathways were significantly downregulated, while 10% (10) of the pathways were upregulated. Sixty-two percent (25) of the pathways were significantly downregulated in oligodendrocytes, while only 4% (1) of the pathways were upregulated. Significant changes in pathway expression were not detected in Gad2 + interneurons and medium spiny neurons, although both showed a trend toward upregulation across most of the molecular pathways. Twenty percent (8) of the pathways were significantly upregulated in dopaminergic neurons, while none were significantly downregulated; however, the remaining pathways overwhelmingly trended toward upregulation (Figure 4A). The most significantly altered pathways in microglia were those related to phagocytosis and autophagy (Figure 4A).

BDNF and irisin are peptides whose expression in the brain increases with exercise. Western blots for those peptides revealed a 5-fold increase in BDNF in basal ganglia homogenate of rats infused with eExosome compared to sedentary exosome control and a 3.4-fold increase in irisin in the eExosome-treated rats compared to control (Figure 4B).

## Discussion

This proof-of-concept study demonstrated for the first time that some effects of exercise that may contribute to brain health are transferable and partially driven by exosomes from plasma. Exosomes extracted from either healthy exercising or healthy sedentary donors were infused into the brains of aged sedentary male rats for 7 consecutive days. The exosomes were validated, and their RNA content was sequenced in a companion study (Citron et al., 2025). In the previously study, 27 RNAs were upregulated in eExosomes compared with controls, including Rgs4, Npy, and mt-Atp6. We hypothesize that these mRNAs are responsible for providing neuroprotective changes and driving exercise-adaptive responses. Rats treated with eExosomes abundant in Rgs4, Npy, and mt-Atp6 have an increase in processes such as autophagy, cAMP signaling, axon guidance, and calcium signaling (Figure 4A). An increased expression of Rgs4 increases cAMP signaling with brain region-specific outcomes (Ni et al., 1999). NPY participates in several mechanisms and has been shown to stimulate autophagy (Aveleira et al., 2015) and axonal guidance (Hokfelt et al., 2008). Loss-of-function mutations in genes coding for mt-Atp6 (Leigh syndrome) result in mitochondrial dysfunction and calcium dysregulation (Galera-Monge et al., 2020). Conversely, increased levels of mt-ATP6 could lead to an increase in calcium signaling, as observed in this study (Figure 4A). DMT1 is upregulated in PD patients and in the MPTP-induced mouse model of PD. DMT1 upregulation coincided with iron accumulation, increased ROS, and neurodegeneration, while expression of the impaired mutant form of DMT1 provided neuroprotection (Salazar et al., 2008).

No adverse events were detected during or after the procedure, suggesting that the transfer of exosomes between donors and recipients is safe even with exosomes harvested from plasma and transferred into the brain. The results of this study revealed changes in specific glial and neuronal signaling pathways (Figure 4A). Induction of brain peptides known to be produced with exercise, BDNF and irisin, was also observed (Figure 4B). Together, these findings indicate that the effects and potential benefits of exercise can be safely transferred from healthy active donors to sedentary aged recipients.

### Exercise plasma exosomes reduce glial activity overall but promote autophagy

Inflammaging is a general propensity of the brain to become more pro-inflammatory with advancing age (Franceschi et al., 2000). Total numbers and reactivity of astrocytes (Palmer and Ousman, 2018) increase with age, while microglia increase in their reactivity but maintain the same total numbers throughout life (Antignano et al., 2023). An overall increase in the abundance of pro-inflammatory cytokines, such as interleukin-1β, also accompanies aging. These pro-inflammatory changes in astrocytes and microglia have been associated with deteriorating synaptic function (Lynch, 2010). In an aged rat stroke model, an exaggerated pro-inflammatory response predicted poor outcomes (Dinapoli et al., 2010). Oligodendrocytes are important for myelination, and they also provide metabolic support to neurons, but recent studies suggest that they are also an important nexus in communication between microglia and neurons (Green et al., 2025). They can release pro-inflammatory cytokines and promote neurodegeneration (Castelo-Branco et al., 2024). Our findings reveal a global downregulation of several molecular pathways in glial cells, including oligodendrocytes, astrocytes, and microglia (Figure 4A). The most pronounced change was observed in the oligodendrocytes, in which 65% of the identified pathways were significantly downregulated, while only one pathway, for aminoacyl-tRNA biosynthesis, was upregulated. Defects in this pathway, including mutations in aminoacyl-tRNA synthetase, result in defective myelination (Turvey et al., 2022), and its upregulation in our study, specifically in the oligodendrocytes, suggests that the infusion of exosomes may promote myelination. This pathway, however, is reduced in astrocytes, in which we also observed the suppression of the myelination and myelin maintenance pathways. Conversely, astrocytes activated motor protein and cytoskeleton assembly pathways, suggesting increased cell motility or cellular transport. eExosomes also suppressed pathways associated with gliogenesis and ERK1/MAPK signaling in the astrocytes. In the microglia, exosomes activated cytokine and SMAD transduction, suggesting increased TGFβ signaling, while suppressing Wnt and other cellular proliferation pathways. Decreases in reactive and proliferation pathways, combined with the overall suppression of molecular pathways in astrocytes and microglia, suggest that even a weeklong infusion of eExosomes may significantly reduce glial bioactivity across multiple pathways (Figure 4A). This reduction in glial activity may contribute to improved functional outcomes that result from exercise. Autophagy molecular pathways were upregulated in microglia (Figure 4A), and their activation is strongly associated with exercise (He et al., 2012). Moderate treadmill exercise has been shown to enhance microglial autophagy during the early stages of neurodegeneration, which may improve the clearance of protein aggregation associated with age-related neurodegenerative diseases (Gao B. et al., 2025). Notably, glia are dynamic, and these results were obtained from tissue within 30 min of the last infusion and may be transient in nature. Reduced metabolic activity may also be interpreted as a response to stress. Longer endpoints after cessation of treatments would help resolve these findings.

### An increase in neuroprotective peptide levels and dopaminergic neuron pathway activity may partially explain the protective effects of moderate exercise in slowing PD

In our companion study, citron et al, Citron et al. (2025), we showed that moderate treadmill exercise slowed dopaminergic neuron loss in a rat preclinical model of PD. In this study, we found increases in BDNF and irisin, peptides known to promote neuronal survival in basal ganglia enriched homogenate (Figure 4B), after infusion with plasma exosomes derived from exercising donors. A decrease in gene expression for mitochondrial complexes I and III and PERK was also observed after infusion together with *CAMKII* upregulation in dopaminergic neurons (Figures 3AB). These results are consistent with known exercise-induced enhancement of CAMKII expression, which can subsequently increase BDNF expression (Vaynman et al., 2007). Moderate exercise transiently increases ROS production in neurons, which leads to compensatory responses; in this case, it decreases the expression of complexes I and III that generate ROS. Transient downregulation or bypass of mitochondrial electron transport chain complexes was reported in adipose (Esbjornsson et al., 2024) and muscle (Nilsson et al., 2019) tissue after intense exercise. These complexes are the primary sites of intracellular ROS production. Decreases in complexes I and III would lower adenosine triphosphate (ATP) production and, in turn, signal to the cell to upregulate the expression of neuroprotective mechanisms, including the increased expression of BDNF (Quan et al., 2020). Complex I, complex III, and PERK genes are involved in PD-related pathways, and eExosomes could be modifying their expression in an exercise-like neuroprotective manner. Oxidative phosphorylation, although upregulated, was not statistically significant and was accompanied by a decrease in pyruvate metabolism. These changes could be interpreted as ultimately stressful to the cell since an increase in energy production through an increase in oxidative phosphorylation may not be met due to a decrease in pyruvate metabolism. However, the totality of significantly upregulated dopaminergic pathways was trophic, e.g., axon guidance, neuroactive ligand signaling, and calcium signaling pathway (Figure 4A).

Regular exercise modulates iron homeostasis and was found to significantly downregulate DMT1 mRNA levels in Alzheimer’s disease model mice (Belaya et al., 2021), similar to what we reported in sedentary rat dopaminergic neurons after only 1 week of eExosome treatment (Figure 3B). Treatments with BDNF have been shown to decrease both mRNA and protein levels of DMT1 *in vitro* (Zhang H. Y. et al., 2014). These findings indicated that exercise may improve neuronal survival by correcting iron trafficking dysregulation, also known to occur in PD patients and in animal models of PD.

### Study limitations

Polymer-based exosomal precipitation may co-isolate non-exosomal components such as apoptotic bodies. ICV infusion was performed for 7 days; longer infusions may have produced more significant individual gene expression changes. Although significant, immunoblot should be interpreted with caution due to small sample size. Isolated exosomes were not probed for (negative) biomarkers of nuclear, mitochondrial, or golgi subcellular origin and this limitation will be addressed in future studies.

### Future direction

Future studies will test longer intravenous infusions of exosomes in sex-balanced groups to determine brain penetrance and functional outcomes in aged and PD models to demonstrate clinically relevant outcomes. To understand the contribution of individual RNAs identified in our companion study (Citron et al., 2025), RNAs will be delivered individually or in tandem using synthetic exosomes or lipid nanoparticles. These studies will identify RNA combinations most effective at inducing neuroprotective exercise-like adaptations for translational applications.

## Data Availability

The datasets presented in this study can be found in online repositories. The names of the repository/repositories and accession number(s) can be found at: https://www.ncbi.nlm.nih.gov/geo/query/acc.cgi?acc=GSE326987.

## Footnotes

1
https://genomebiology.biomedcentral.com/articles/10.1186/s13059-019-1874-1
